# Sustainable Use of Nanomaterials in Textiles and Their Environmental Impact

**DOI:** 10.3390/ma13225134

**Published:** 2020-11-13

**Authors:** Haleema Saleem, Syed Javaid Zaidi

**Affiliations:** Center for Advanced Materials (CAM), Qatar University, Doha 2713, Qatar; haleema.saleem@qu.edu.qa

**Keywords:** nanomaterials, environmental impacts, textiles, toxicity, health and safety

## Abstract

At present, nanotechnology is a priority in research in several nations due to its massive capability and financial impact. However, due to the uncertainties and abnormalities in shape, size, and chemical compositions, the existence of certain nanomaterials may lead to dangerous effects on the human health and environment. The present review includes the different advanced applications of nanomaterials in textiles industries, as well as their associated environmental and health risks. The four main textile industry fields using nanomaterials, nanofinishing, nanocoatings, nanofibers, and nanocomposites, are analyzed. Different functional textiles with nanomaterials are also briefly reviewed. Most textile materials are in direct and prolonged contact with our skin. Hence, the influence of carcinogenic and toxic substances that are available in textiles must be comprehensively examined. Proper recognition of the conceivable benefits and accidental hazards of nanomaterials to our surroundings is significant for pursuing its development in the forthcoming years. The conclusions of the current paper are anticipated to increase awareness on the possible influence of nanomaterial-containing textile wastes and the significance of better regulations in regards to the ultimate disposal of these wastes.

## 1. Introduction

The idea of nanotechnology was initially presented by Richard Feynman in the year 1959, through his speech “There’s Plenty of Room at the Bottom”, which was delivered at an American Physical Society conference, at the California Institute of Technology (Feynman, 1959, [1]). The concepts introduced by Feynman were unobserved until 1974, when Norio Taniguchi presented the term “nanotechnology” (Taniguchi, 1974, [2]). The term “nano” means one-billionth or 10^−9^ henceforth, one nanometer is referred to as one billionth of a meter. Currently, an extensive range of fabrication systems are present that are capable of controlling and producing nanostructures to the preferred composition, size, morphology, shape, and crystalline structure. The two typical fabrication methods utilized are “top-down” and “bottom-up”. These days, nanotechnology contributes to the prospective opportunities in developing improved materials with advanced properties for utilization in different application fields. The atoms inside nanoparticles are perfectly ordered and consequently, while the material dimensions change from macro-size to nano-size, extensive variations happen in the material properties (Yang et al., 2019, [3]).

At the present time, researchers can develop various nano-sized materials such as nanoclays, carbon nanotubes, nanofibers, and graphene with lighter, stronger, increased chemical reactivity, and more prolonged control on the light spectrum (Khan et al., 2019, [4]). An improved understanding of the properties of nanomaterials provides a way for developing progressive materials in the upcoming years with the probability for improving the life quality. Nanomaterials are gradually turning out to be commercialized, starting to progress as commodities, and used in numerous advanced technological applications and products, including a wide variety of consumer products. The design as well as preparation of nanomaterials with a unique combination of textile material is anticipated to expand the demanding scope in the future (Verma et al., 2020, [5]).

At present, the engineered nanomaterials are being examined extensively by research institutes as well as industries for improving prevailing functions in products together with implementing new ones. Regardless of such developments in nanomaterial technology, data regarding the probable effects of nanomaterials on human health and the environment has been inadequate until now (Kumar et al., 2018, [6]) (Mishra et al., 2018, [7]), (Kumar et al., 2018, [8]). Nanosafety is an increasing concern as exposure to engineered nanomaterials has been related to several health effects inclusive of carcinogenicity, genotoxicity, pulmonary inflammation, and circulatory effects (Leong, 2017, [9]), (Johnston et al., 2020, [10]), (Mirshafiee et al., 2018, [11]), (Karim et al., 2020, [12]), (Dobrovolskaia et al., 2013, [13]). Due to the fact that the nanomaterials may not be recognizable subsequent to its discharge into the surroundings, these materials could cause various kinds of ecological problems as long as the remediation scheme is unsafe. The data acquisitions on emission as well as ecological concentrations of nanomaterials of the nanotextiles is extremely important. Subsequently, additional study is crucial for scientifically describing the structure-function relation of nanomaterials with respect to the fundamental chemistry (as an illustration, functionality and toxicity). Additionally, comprehensive risk assessments should be performed on nanomaterials that present an actual exposure danger throughout its fabrication or usage (Ahmad et al., 2020, [14]), (Kawai et al., 2019, [15]), (Auffan et al., 2019, [16]), (Oomen et al., 2018, [17]), (Schulte et al., 2018, [18]). Henceforward, green nanoscience has been suggested for reducing the probable environmental threats and human health risks from the fabrication and usage of nanomaterials and to develop the substitution of prevalent items with progressive nanomaterials that are more eco-friendly (Iavicoli et al., 2014, [19]), (McKenzie et al., 2004, [20]), (Hutchison et al., 2008, [21]), (Bamoharram et al., 2011, [22]). In this review paper, we discuss the application of nanomaterials in the textile industries. These sorts of studies may be advantageous for the suitable advancement of applications and research interest towards the further development of nanomaterials. To the best of our knowledge, there are not many works about the state-of-the-art progress in nanotechnology for application in textile industries. This review paper highlights the sustainable use of nanomaterials in textiles, their release from textiles, and the different approaches for examining nanomaterial toxicity. In addition, we have comprehensively studied the hazardous effects of textile field nanomaterials on human health and the environment.

## 2. Application of Nanomaterials in Textile Industry

**Figure 1:**
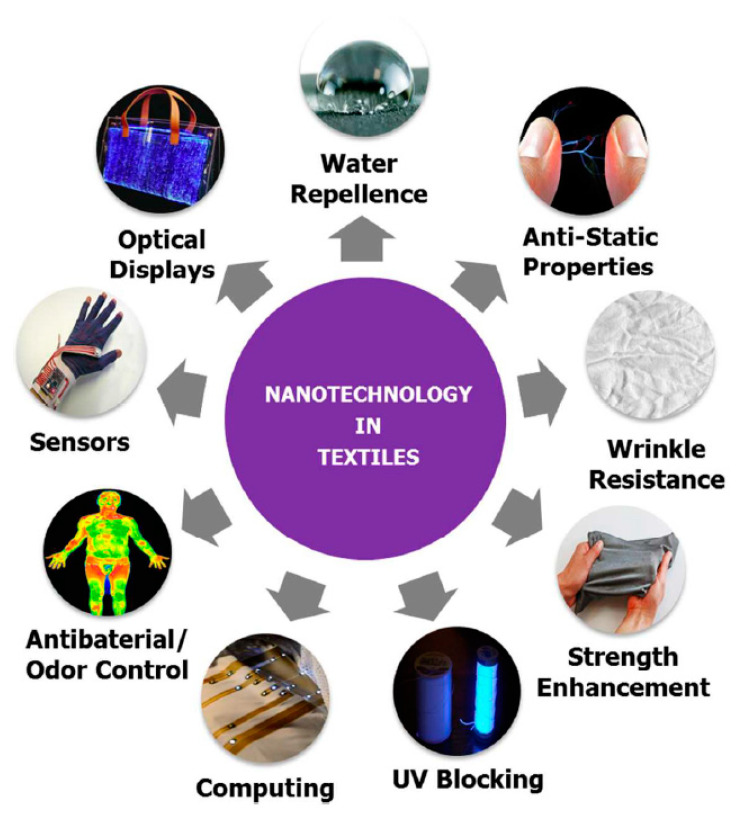
A diagrammatic representation of various utilizations of nanotechnology-based textiles. Reproduced from reference (Yetisen et al., 2016, [56]).

Presently, the textile industry is a significant user of nanotechnology and there are a remarkable number of nanotextiles present in the market, inclusive of several consumer goods, which includes nanomaterials (Karst, D et al., 2006, [23]), (Jatoi et al., 2021, [24]), (Darwesh et al., 2021, [25]), (Schoden et al., 2021, [26]), (Yilmaz, 2018, [27]), (Ehrmannet al., 2020, [28]), (Abdullaeva, 2017, [29], (Riaz et al., 2019, [30]). Nanotextiles are regarded as conventional textiles with the inclusion of nanomaterials. These advanced textiles offer different functionalities like flame retardancy, self-cleaning, dirt repellency, water repellency, ultraviolet radiation protection, or antibacterial property (Almeida et al., 2017, [31]), (Brown et al., 2007, [32]), (Radetic et al., 2013, [33]), (Ibrahim et al., 2015, [34]), (Sundarrajan et al., 2010, [35]), (Afzali, A. et al., 2016, [36]), (Sharon et al., 2019, [37]), (El-Naggar et al., 2018, [38]), (Xue et al., 2020, [39]), (Gadkari et al., 2020, [40]), (Elsayed et al., 2020, [41]), (Mejia et al., 2017, [42]). Nanocoatings and nanofinishings are enhancing the possible utilizations of textile materials in different fields (Banerjee et al., 2019, [43]), (Jadoun et al., 2020, [44]), (Gokarneshan et al., 2017, [45]), (Perera et al., 2013, [46]), (Ferraris et al., 2014, [47]). The usage of nanofibers and nanocomposite based coatings/finishings have demonstrated a huge possibility in emerging functional and high-performance textiles (Bashari et al., 2018, [48]), (Riaz et al., 2018, [49]), (Haque et al., 2019, [50]), (Lund et al., 2018, [51]), (Silva et al., 2019, [52]), (Shabbir et al., 2020, [53]), (Ul-Islam et al., 2018, [54]). The study by (Singh et al., 2020 [55]) reviewed the latest studies involving the modification and characterization of textile, highlighting plasma and nano-pretreatment. Figure 1 diagrammatically presents various nanotechnology-enhanced textiles. Due to its higher surface area to volume ratio and nanoscale dimensions, the nanomaterials have increased potential for providing different functionalities in the textiles. Various nanomaterials that are utilized for textile utilization are mostly: (1) Carbon-based nanomaterials such as graphene, carbon nanofibers, and carbon nanotubes; (2) inorganic nanoparticles such as metal oxide, metal, and nanoclay; (3) core-shell nanoparticles; (4) composite nanomaterials; (5) hybrid nanomaterials; and (6) polymeric nanomaterials. Table 1 provides information on the different nanomaterials most frequently utilized for functionalization in textiles.

### 2.1. Innovations in Nanotechnology-Based Textile Industry Applications

The four main fields through which nanomaterials and nanotechnology find utilizations in the textile industry are analyzed below.

#### 2.1.1. Nanofinishing

Nanofinishing is the process in which the colloidal solution or ultrafine dispersion of nanomaterials is applied to a textile material for enhancing some of the functionalities (Joshi et al., 2018, [57]), (Haji et al., 2016, [58]), (Ghosh et al., 2018, [59]), (Radetic et al., 2019, [60]). It has certain benefits over traditional finishing, which are principally: (i) In general, in the case of nanofinishing, it only requires a lesser quantity of nanomaterials relative to bulk materials utilized in traditional finishing, for obtaining a similar effect. (ii) They do not influence the aesthetic feel of the textile materials. (iii) These nanofinishings are more durable due to the increased surface area-to-volume ratio of nanomaterials along with its homogenous distribution in textile material. (iv) Certain functionalities that are hard to attain by traditional finishes could possibly be developed by nanofinishing (Gokarneshan et al., 2018, [61]), (Ghosh et al., 2020, [62]). For almost two decades, the nano-finishing of cellulose textile material while using copper and copper oxide nanoparticles has been in the focus of science and textile industries (Radetic et al., 2019, [63]).

#### 2.1.2. Nanocoating

In a nanocoating process, a thin layer approximately less than 100 nm thickness is deposited on the substrate in order to improve certain properties or for contributing advanced functionality. Traditional coatings possess certain disadvantages such as (i) less durability, (ii) poor abrasion resistance, (iii) strength loss, (iv) less flexibility, and (v) improper adhesion between the substrate and coating layer (Nguyen et al., 2018, [64]). The aforementioned problems of traditional coatings could be solved by the utilization of nanocoatings (Joshi et al., 2011, [65]), (Smole et al., 2006, [66]), (Peng et al., 2019, [67]). The nanomaterial coating on fabrics will not influence their breathability or hand-feel (Temesgen et al., 2018, [68]).

#### 2.1.3. Nanofibers

**Figure 2:**
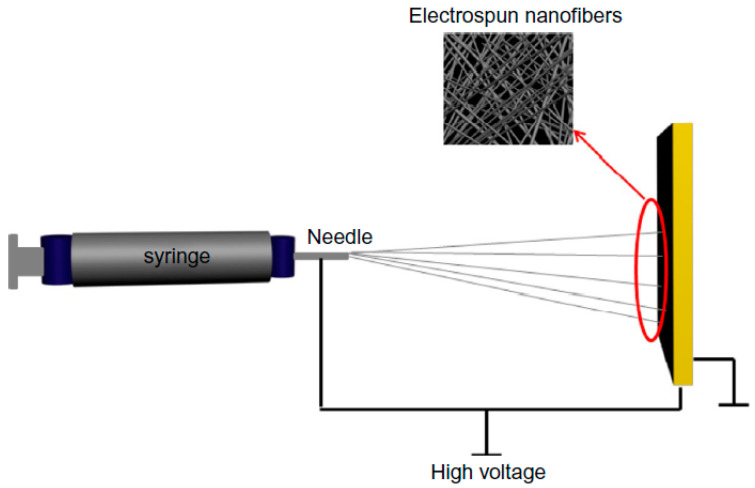
Electrospinning design. Reproduced from (Montazer et al., 2018, [74]).

**Figure 3:**
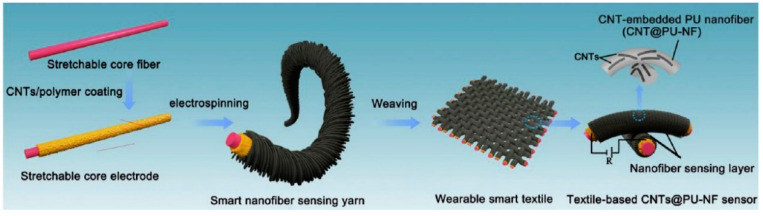
Schematic of the fabrication processes of carbon nanotube-incorporated nanofiber sensing yarn. Reproduced from (Qi et al., 2020, [72]).

For the fabrication of nanofibers, different techniques are used, like electrospinning (Figure 2), self-assembly, force spinning, melt blowing, and island-in-sea (bicomponent nanofiber) (Almetwally et al., 2017, [69]), (Nayak et al., 2019, [70]), (Naeem et al., 2019, [71]). Out of these techniques, the electrospinning is considered the most convenient one due to its low cost, higher rate of production, higher porosity, and ability to control nanofiber morphology and diameter. A stretchable piezo-resistive carbon nanotube-incorporated nanofiber sensing yarn (Figure 3) was first designed as well as prepared by a facile electrospinning technique by (Qi et al., 2020, [72]). Moreover, the nanofibers are unique with high capability as active layers in face masks, to protect people against diseases such as coronavirus (Tebyetekerwa et al., 2020, [73]).

#### 2.1.4. Nanocomposites

**Figure 4:**
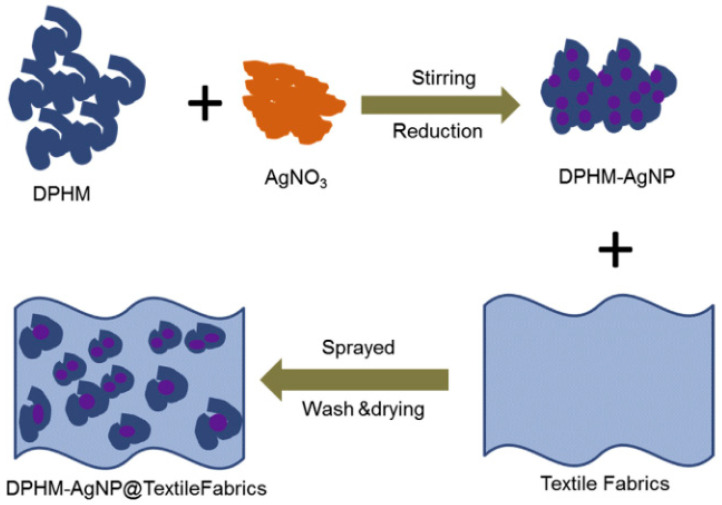
Schematic diagram showing the synthesis of diphosphate malonate-silver nanoparticle (DPHM-AgNP) nanocomposites and their treatment on textile fabrics. DPHM: Diphosphate malonate, AgNO_3_-Silver nitrate. Reproduced from (Attia et al., 2016, [76]).

Nanocomposite is a multiphase solid material in which the minimum one dimension of the reinforcing phase is in nano-level (Malhotra et al., 2017, [75]). In the case of polymer-based nanocomposites, nanomaterials are dispersed in polymer matrices. The polymer nanocomposite-based coatings and fibers have enormous possibility in the production of functional as well as superior-performance textiles. Novel nanocomposite materials have been developed using facile one pot method. In a study by (Attia et al., 2016 [76]), the team developed nanocomposites based on silver nanoparticles as well as diphosphate malonate (DPHM) as organic phosphates (Figure 4). The mass ratios of both organic phosphate and silver nanoparticles in the nanocomposites were varied as well as optimized. Various textile fabrics have been treated with the nanocomposites developed to improve their fire retardancy and antibacterial properties. It was found that the flame retardancy of the treated textiles increased significantly.

### 2.2. Functional Textiles with Nanomaterials

In the following section, we discuss nanomaterial included different functional textiles, such as flame-retardant textiles, UV Protective textiles, antimicrobial and antibacterial textiles, water and oil repellent textiles, anti-odor textiles, wrinkle resistance textiles, and antistatic textiles. The study by (Vigneshwaran et al., 2020 [83]) demonstrated the fundamental mechanisms involved in the usage of nanomaterials for contributing the functional properties in cotton textiles, applicable issues as well as future scope for commercial utilization.

#### 2.2.1. Flame-Retardant Textiles

Material scientists are planning to replace traditional flame-retardant systems with eco-friendly substitutes like the utilization of nanotechnology or flame retardant nano-additives (Attia et al., 2016, [76]), (Erdem et al., 2009, [84]), (Attia et al., 2017, [85]), (Shariatinia et al., 2015, [86]), (Kundu et al., 2020, [87]), (Rivero et al., 2015, [88]), (Ortelli et al., 2019, [89]), (Ortelli et al., 2018, [90]), (Carosio et al., 2012, [91]), (Saleemi et al., 2020, [92]), (Ali et al., 2020, [93]), (Butola et al., 2020, [94], (Li et al., 2018, [95], (Sharma et al., 2018, [96]). (Norouzi et al., 2015 [97]) studied the influence of nanoparticles along with standard flame retardants on the flame retardation of several textile polymers. The results demonstrated that a majority of the nanoparticles could enhance the flame retardant properties and thermal stability of the textile polymers. The foremost mechanisms involved are the development of a shielding barrier layer consolidated with char promotion as well as free radical trapping. However, the level of improvement depends on numerous parameters like the composition and morphology of the nanoparticle, migration speed of nanoparticles to the surface, dispersion of the nanoparticles in the polymer matrix, and compatibility between polymer and nanoparticle.

In the work by (Yazhini et al., 2015, [98]), the team developed crosslinked cotton coated with nanocomposites (Polypyrrole-zinc oxide and polypyrrole-zinc oxide-carbon nanotube) for ultraviolet-protection as well as flame retardant finishes. It was confirmed that the polypyrrole-Zinc oxide-carbon nanotube composite-coated cotton was noted to demonstrate improved properties relative to uncoated cotton. (Fanglong et al., 2016, [99]) developed flame retardant mixtures of traditional intumescent flame retardant and nanosilica, and these were applied onto cotton fabric for investigating the synergistic influence of nano-silica on the fire resistance as well as thermal stability of the intumescent flame retardant system. The test results showed that a suitable inclusion of nano-silica into the conventional intumescent flame retardant system could enhance the fire protection properties of cotton fabric- intumescent flame retardant system to a definite extent, however it led to a reduction in the thermal stability of the system.

#### 2.2.2. UV Protective Textiles

Currently, because of the depletion of the ozone-layer in the atmosphere, ultra-violet radiation is entering the surface of the earth, which is noted to have a harmful effect on both clothes and on human skin. The increased exposure to ultraviolet radiation enhances the probabilities of having several toxic diseases such as skin cancer. Consequently, protection against ultraviolet radiation has turned out to be a necessary property for clothing and textiles (Dhineshbabu et al., 2019, [100]). Certain metal oxide nanomaterials like magnetite nanoparticles, titanium dioxide nanoparticles, zinc oxide nanoparticles, and nano-ceria successfully block ultraviolet radiations, guaranteeing a sustainable as well as better performance relative to the organic ultraviolet absorbers (Sedighi et al., 2018, [101]), (Kathirvelu et al., 2009, [102]), (Becheri et al., 2008, [103]), (Fouda et al., 2018, [104]), (Tsuzuki et al., 2010, [105]), (Cakir et al., 2012, [106]), (Farouk et al., 2010, [107]), (Radetic et al., 2013, [108]), (Attia et al., 2017, [109]), (Attia et al., 2017, [110]). In recent times, the aforementioned nano-inorganic-ultraviolet additives are commonly preferred instead of the organic ones due to their exceptional properties such as harmlessness and chemical stability under UV radiation as well as higher temperature exposure. Properties such as particle size, phase composition, surface properties, crystallinity, and crystal structure are different factors that influence the ultraviolet blocking property of nano-sized ultraviolet additives (Lee, 2009, [111]), (Dhineshbabu et al., 2018, [112]).

In the latest research work by (Noorian et al., 2020, [113]), zinc oxide nanoparticles were in situ prepared on the modified cotton fabric for developing the multifunctional fabrics. This zinc oxide-4-aminobenzoic acid ligand oxidized cotton fabrics demonstrated superior ultraviolet-protection and substantial antibacterial effectiveness subsequent to 100 abrasion cycles and 20 washing cycles, and hence this could be used in innovative protective textiles. (Dhineshbabu et al., 2019 [100]), designed ultraviolet-blocking as well as fire resistant cotton fabric by coating polyurethane-based MnO_2_-FeTiO_3_ nanocomposites. The MnO_2_-FeTiO_3_ coated cotton fabrics showed a durable ultra-violet blocking capability and presented better fire resistant properties evaluated utilizing the limited oxygen index. Additionally, the coated cotton fabric maintained its properties in spite of 10 water-laundering cycles thus contributing smart, sustainable, and durable fabric for protective clothing utilization.

#### 2.2.3. Antibacterial and Antimicrobial Textiles

**Figure 5:**
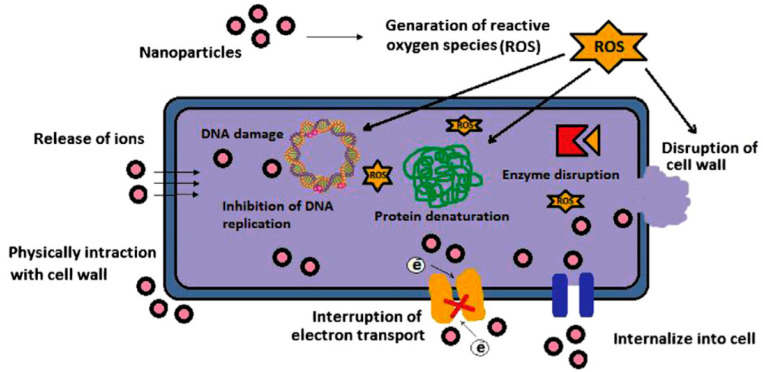
Different mechanisms of antimicrobial activity of metal-oxide and metal nanoparticles. Reproduced from (Dizaj et al., 2014, [126]).

**Figure 6:**
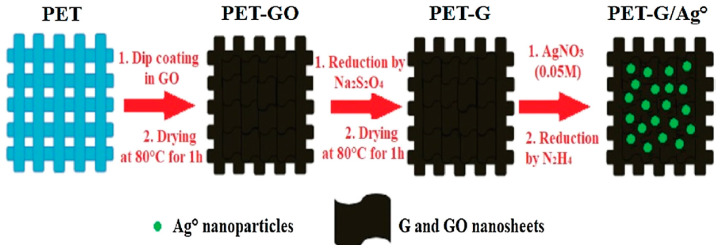
Process for the preparation of multifunctional polyester fabric coated by graphene/silver nanoparticles. Reproduced from (Ouadil et al., 2019, [125]).

Textile fabrics, particularly ones made up of cellulose fibers like lyocell, viscose, linen, and cotton have a greater tendency to be harmed by microorganisms, for example, protozoa, algae, fungi, virus, and bacteria, in the course of their service life (Ahmed et al., 2017, [114]), (Bu et al., 2019, [115]), (Hebeish et al., 2011, [116]), (Xue et al., 2012, [117]), (Zhang et al., 2009, [118]), (Budama et al., 2013, [119]), (Liu et al., 2014, [120]), (Perelshtein et al., 2008, [121]), (Zhang et al., 2014, [122]), (Attia et al., 2017, [123]). Recently, because of the enhancement in awareness about hygiene and health, the antimicrobial feature has developed into an essential prerequisite for all clothes, medial textiles, and household products. In recent times, various metal oxide (like copper oxide, zinc oxide, and titanium dioxide) and metal (such as silica, titanium, gold, zinc, copper, and silver) nanoparticles are receiving considerable research attention as prospective antimicrobial agents. Nanomaterials with a higher surface area-to-volume ratio contribute a superior antimicrobial characteristic relative to traditional antimicrobial agents. Figure 5 demonstrates different mechanisms of antimicrobial activity of metal-oxide and metal nanoparticles. Table 2 presents the textiles modified using different nanoparticles for antimicrobial effects. The utilization of nanocomposites of antimicrobial agents in textiles showed a positive synergistic antimicrobial property relative to a single nanomaterial. Economical and ecofriendly antibacterial properties of cotton fibers loaded with silver nanoparticles prepared from natural Chinese Holly plant extracts were studied by (Ullah N et al., 2014 [124]). The generation of silver nanoparticles from Chinese Holly plant extracts were noted by UV–vis spectrophotometer and noted to be less than 100nm in size, as confirmed by electron microscopy analysis. The antimicrobial properties of these cotton fibers incorporated with silver nanoparticles were assessed against gram-negative *Escherichia coli* bacteria. The test results confirmed superior antibacterial properties by incorporating 1.5% to 4.5% of Chinese Holly leave extracts. The cotton fibers also illustrated fine antibacterial efficacy after numerous washings, making it appropriate for medical usages with an ease. The process for the preparation of multifunctional polyester fabric coated by graphene/silver nanoparticles is shown in Figure 6 (Ouadil et al., 2019, [125]).

#### 2.2.4. Water and Oil-Repellent Textiles

Water and oil repellency has turned out to be a requirement for entire clothes and this has developed into one of the main targets for textile manufacturers and scientists for years (Asif et al., 2018, [132]). Presently, advanced nanocoatings or nanofinishings are satisfying a majority of similar market necessities with oil and water repellent or superhydrophobic textiles. Information on nanotechnology and textile mutually assist to progress an advanced conception of ‘self-cleaning textiles’, in which the textiles possess an ability to be cleaned with no laundry treatment (Katiyar et al., 2020, [133]), (Montazer et al., 2020, [134]). There exist two diverse methods prevalently utilized for the advancement of self-cleaning textiles, which are: (i) Photocatalytic activity and (ii) the lotus effect.

The lotus effect is produced by the surface modification of the textile fabric by nanocoating or nanofinishing, usually by utilizing surface modified carbon nanotubes, zinc oxide nanorods, nano-zirconia, or nano-silica (Das et al., 2015, [135]), (Joshi et al., 2012, [136]). In the case of the photo-catalytic activity method, zinc oxide or titanium dioxide nanoparticle-based coating or finish formulations are utilized to develop self-cleaning textiles. Titanium dioxide’s photocatalytic activity is dependent on the crystal framework, and the anatase grade titanium dioxide demonstrated superior photo-catalytic activity against contaminants and colorants. (Wang et al., 2010 [137]) demonstrated that gold/titanium dioxide/silicon dioxide nanosol is a superior photocatalyst relative to titanium dioxide nanosol, displaying an improved self-cleaning property also in the existence of visible light. There are also several other works that demonstrate the cotton fabric’s photocatalytic self-cleaning property by treatment with titanium dioxide nanowire and titanium dioxide nanowire doped Ag-PVP (Hebeish et al., 2013, [138]), graphene/TiO_2_ nanocomposites (Karimi et al., 2014, [139]), etc. The work by (Landi et al., 2019 [140]) reported the preparation of photocatalytic nanocomposite materials based on nitrogen-doped nano-titanium dioxide, silicon dioxide, and various percentages of HY zeolite. It was noted that the fabrics coated with the photocatalysts, demonstrated similar RhB decolorization (almost 95%) after 5 h. Fluorine-free superhydrophobic cotton fabrics, having the self-cleaning photocatalytic ability, were fabricated by the combination of superhydrophobic SiO_2_ and photoactive titanium dioxide (Xu et al., 2015, [141]).

#### 2.2.5. Anti-Odor Textiles

Tourmaline nanomaterial-based nanofinishing on textiles contributes to an odor-resisting property and has the ability to separate till 75% sticky moisture, 99.99% of bacteria, and 90% of odor (Joshi et al., 2019, [142]). The inclusion of fragrant material (aroma) in antiodor/antimicrobial finishing by nanoencapsulation in synthetic fibers or by formulation might assist to release fragrance in the course of its utilization (Priyadarshinirajkumar et al., 2015, [143]).

#### 2.2.6. Wrinkle Resistance Textiles

Cotton fabric is extremely vulnerable to developing creases at the time of usage. In traditional techniques, resin-based finishings are commonly utilized for imparting the wrinkle resistance feature to textile fabrics. The utilization of nanoparticles such as silicon dioxide and titanium dioxide have the ability to overcome certain restrictions to traditional crease resistant finish (Haque et al., 2019, [144]), (Tripathi et al., 2019, [145]). The study by (Hezavehi et al., 2015, [146]) evaluated the wrinkle behavior as well as wrinkle resistance ability of cotton fabrics dyed using Direct Blue 2B in the absence and presence of titanium dioxide nanoparticles. The test results confirmed that the wrinkle-resistance property of cross-linked fabrics was enhanced subsequent to direct dyeing. (Uğur et al., 2017 [147]) investigated the advanced flame retardant, wrinkle resistant, and durable finishing of linen by utilizing 1,2,3,4-Butanetetracarboxylic acid, nano-polyurethane for crosslinking process, and aluminum oxide nanoparticles for catalyst in the padding procedure. It was found that the flax fabrics applied with the aluminum oxide nanoparticles showed improved flame retardant and wrinkle resistance properties.

#### 2.2.7. Antistatic Textiles

Due to increased moisture content, cellulose fibers like lyocell linen, viscose, cotton, etc. do not mount up the static charge. On the other hand, synthetic fibers like nylon are susceptible to the generation of static charge due to lower moisture regain. Consequently, several studies have been performed on the development of anti-static textiles by the incorporation of certain conducting nano-fillers (Memon et al., 2018, [148]). Titanium dioxide nanoparticle, nano-silver, zinc oxide nanoparticle, antimony-doped tin oxide, and silane nanosol can be used to provide anti-static ability in synthetic fibers (Zhang et al., 2009, [149]), (Hassan et al., 2019, [150]), (Hossain, M et al., 2013, [151]), (Yadav et al., 2006, [152]). Due to the fact that the aforementioned nanomaterials are conductive in nature, these materials dissipate static charges mount up on the surface of fiber.

## 3. Nanomaterials in Textile Industries—Environmental, Health, and Safety Concerns

Textile material is considered to be our second skin for an entire day. We use textiles for aesthetic, protective, decorative, and several other applications. Most textile materials are in direct and prolonged contact with our skin. Consequently, the influence of carcinogenic and toxic substances that are available in textiles must be comprehensively examined. One of the most modern groups of prospective dangerous substances belongs to the group of engineered nanomaterials. The significance of engineered nanomaterials has been acknowledged by the textile sector, due to the fact that these materials have the ability to modify the chemical and physical properties of textile materials and textile fibers—improve water and stain resistance, enhance the capability of materials for absorbing dyes, and alter the wettability depending on surface roughness and surface energy. The aforementioned properties are beneficial for several non-woven and woven textiles like automobile interior fabrics, sportswear, protective clothing, and rainwear (Harifi et al., 2017, [153]), (Shalaby et al., 2020, [154]), (Yu et al., 2018, [155]), (Kausar et al., 2018, [156]). Still there are no adequate studies, however some literature reviews and scientific papers have focused on safety issues related to nanotextiles. Some of the recently published studies include the works by (Rovira eta al., 2019, [157]) and a recent chapter by (Montazer et al., 2018, [158]) in a book entitled Nanofinishing of Textile Materials. Nanotextiles can definitely cause risks for human health, safety, the environment, and sustainability (Kohler et al., 2014, [159]), (Geranio et al., 2009, [160]), (Lorenz et al., 2012, [161]). On the basis of the location of the integration of nanomaterials in textiles, they could be less or more exposed to external influences (Rather et al., 2020, [162]). There have been different studies carried out for assessing the release of silver nanoparticle from antibacterial fabrics into artificial sweat (Wagener et al., 2016, [163]), (Kulthong et al., 2010, [164]), (Kim et al., 2017, [165]), (von Goetz et al., 2013, [166]), (Spielman et al., 2018, [167]), (Stefaniak et al., 2014, [168]), (Balakumaran et al., 2016, [169]), (Milosevic et al., 2014, [170]). (Wagener et al., 2016, [163]) studied the textile functionalization as well as its impacts on the discharge of silver nanoparticles into artificial sweat. Migration tests have been performed for four commercial textiles and for six lab-prepared textiles. Two of these laboratory-prepared textile signifies materials where silver nanoparticles were incorporated inside the textile fiber (composite), while the other laboratory-prepared textile consists of silver particles on the particular fiber surface (coating). The test results confirmed a lesser release of total silver from composites as compared to the surface-coated textile. The particulate portion found inside artificial sweat was noted to be less in the majority of textiles, confirming that the majority of the discharged silver is available as dissolved silver. Additionally, it can be noted that nanotextiles will not discharge more particulate silver, relative to the conventional silver textiles. Moreover, the results confirmed that the functionalization type is the significant parameter influencing the migration.

**Figure 7:**
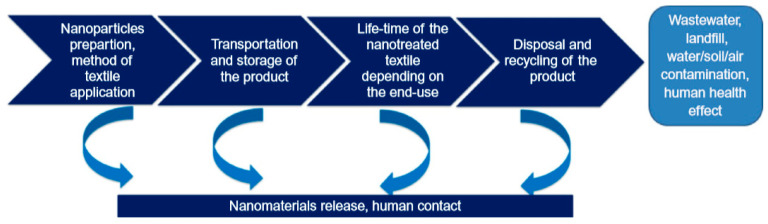
Life-cycle of nanotextiles. Reproduced from (Montazer et al., 2018, [158]).

Despite the aforementioned reported works, attaining wide-ranging information on the risks associated with nanomaterials is still challenging. The aforementioned is because of the range of nanomaterials, manufacture techniques, nanoparticle shape, size, crystallinity, porosity, agglomeration and aggregation, and several other factors. Examination on the dangers associated with nanotextiles and nanofinishing techniques needs a complete understanding of the product’s life-cycle (Figure 7). This take account of the fundamental properties of the nanomaterials, nanomaterial fabrication procedures, and different application techniques utilized for imparting nanocomposites and nanoparticles on the textile substrates or insertion of nanoparticles into fiber formation processing, nanofinishing durability on the surface, storage/transportation of the treated textiles, ultimate product usage, the conditions in which the final product will be exposed to, and product recycling/disposal. Depending on the utilization of the nanotextiles, the disposal of the product will influence soil, water, and commonly the environment, or straight skin contact of human causing threats on the health of a human with ultimate environmental effects. Nanomaterials could be accidentally discharged from the treated samples in the course of their lifetime or exposure of labors to the dangerous effects of nanomaterials might happen at the time of the fabrication process. The life cycle of the product and design of the product regulate the different environmental as well as health exposure situations. As an illustration, the engineered nanomaterials inadvertently discharged from geotextiles might possibly end up in soils, while the engineered nanomaterials involuntarily discharged from T-shirts might make direct contact with humans, finally entering wastewater. Therefore, recommending regulation based on the behavior and effects of nanotextiles needs complete product characterization based on particle stability, surface morphology, shape, porosity, size, chemical composition, as well as propensity to aggregation and agglomeration.

### 3.1. Environmental Risks of Nanomaterials from Textile Industry

A moderately larger fraction of engineered nanomaterials in textiles are assumed to be discharged into wastewater (for example till 20% for silver nanoparticles) (Cucurachi et al., 2019, [171]), (Patnaik et al., 2019, [172]). Nevertheless, the quantity of silver nanoparticles and other engineered nanomaterials accidently discharged into the environment depends greatly on the textile design utilized, particularly how the nanomaterials are incorporated within the textile fiber. Straight discharge of nanomaterials into the air by means of abrasion looks to be of a lesser significance (just 5%). Other flows of nanomaterials from textiles, for example, into waste incineration, are greater from a bulk perspective though, they probably result in just minimal discharges to the surroundings. Therefore, the textiles might constitute a significant source of nanomaterials released into aqueous systems and into soils by the biosolid utilizations. The evaluation of the environmental dangers is extremely dependent on the corresponding product life cycles and on the quantities of engineered nanomaterials manufactured globally. The durability (stability) of the nanomaterials existing in the textile depends on its fabric binding, the influences on the fabric in the course of its life-cycle (manufacture, utilization, disposal/recycling), which could harm the textile material or the bonding between the fibers and nanomaterial, mechanical stress (like pressure, strains), abrasion, temperature changes, high temperatures (till 225 °C in textile finishing), detergents (either during laundry or in textile processing), solvents (during dry cleaning or textile processing), water (washing, rain), body fluids (urine, sweat, and saliva), and ultraviolet radiation. The nano-biocomposite application can be a good substitute to a majority of artificial antibacterial agents because of their biodegradability, increased environmental compatibility, as well as non-toxic nature. Considering a life cycle method for studying the possibility of the release of silver nanoparticles from functionalized textiles, it is possible to find the significance of various phases to silver discharge over time. In a study by (Mitrano et al., 2016 [173]), three distinct lab-prepared nanofabrics were exposed to one or 10 washing cycles under various laundering conditions. It was noted that the total entire metal discharged differed remarkably on the nanoparticle incorporation as well as the washing pattern variant. The test results confirmed that the active landfill environment will not mobilize nanoparticles from the surface of the fabric as easily subsequent to washing relative to the unwashed textile. Increased release of nanoparticles from textile have been noted at the time of the life cycle’s utilization phase instead of the disposal phase.

Despite the fact that nanotextiles have been observed to be efficient in photocatalytic or the absorption degradation of pollutants from impure wastewaters, or the nanofibrous membranes could be employed as air/water filters, there have been several forewarnings in regards to the ecological dangers of nanomaterials. Due to the larger surface area of nanomaterials, these materials are extremely reactive to other materials while discharged during any stages of its life cycle. The aforementioned must be associated with both sides of danger and opportunity as the reaction of nanomaterials with other constituents might contribute to lesser toxicities, thus altering these materials from their unique properties. Conversely, these nanomaterials might carry other harmful materials, generating additional toxicity. If the aggregation/agglomeration of nanomaterials occurs, it is conceivable to separate the nanomaterials from the waste water effluent by filtration or sedimentation techniques. The consequence of sediments on the environment is not totally clear yet. The burning of sewage sludge was reported, even though the whole avoidance of the discharge of nanomaterials has not been demonstrated yet (Som et al., 2011, [174]). Certain nanomaterials could be dissolved in the surroundings producing no consequence, whereas other solutions consisting of metals are extremely harmful to the surroundings. Magnetic separation approaches based on the manufacture of magnetic nanomaterials and their utilization on textile substrates were also established as a capable inexpensive technique for the separation of the nanomaterials from waste water effluents (Harifi et al., 2014, [175]). Fascinatingly, certain engineered nanomaterials might influence the environment less harshly than they might influence human health, whereas the case for some others is vice versa. The aforementioned is specifically true for carbon nanotubes.

**Figure 8:**
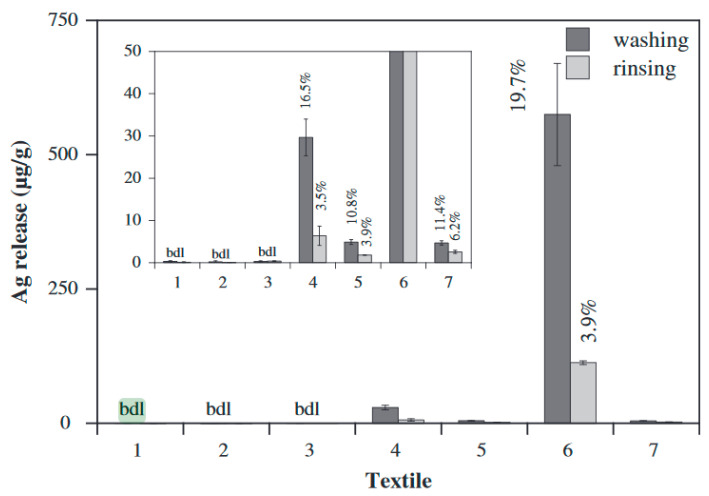
Quantity of silver released from the seven textiles at the time of washing as well as rinsing. The inset presents an expanded outlook of the lower concentration range. The percentage of total silver released is presented on top of the columns. bdl: Below detection limit. Reproduced from (Lorenz et al., 2012, [161]).

The engineered nanomaterials have several benefits depending on the properties, such as less textile laundering because if their antibacterial properties or quick wound healing (Temizel-Sekeryan et al., 2020, [176]). From the analysis of the study by (Lorenz et al., 2012, [161]), four out of the seven silver nanotextiles leached a noticeable amount of silver (Figure 8). Neither the rinsing nor the washing solutions of textiles 1 to 3 contained any silver. In a study by (Piontek et al., 2018 [177]), the team analyzed two Life Cycle Assessment studies on the textile value chain and on the product-service systems. It is good review paper, where the authors presented their findings on the dependence of Life Cycle Assessment studies, lack of accessible information on chemicals, advantages because of the technological development in the area, the significance of the use phase of clothes, and suggest additional routes of research in regards to user behavior as well as methodological development. Merging the conclusions of the two studies helped for the development of a method for conducting the Life Cycle Assessment studies on a product-service system based on existing research as well as research gaps. This could include the development of a method for assessing the environmental benefits when establishing a product-service system.

For the environment, the following criteria were established as critical for the determination of environmental fate as well as effects of engineered nanomaterials (Som et al., 2011, [174]): (1) Certain warning of dangerous effects at existing genuine exposure concentrations, (2) a propensity to be dissolved in water, leading to the vanishing of the engineered nanomaterials, and the development of dissolved metal ions, (3) a propensity for sedimentation or agglomeration under normal conditions, (4) destiny in wastewater facility, and (5) steadiness in the course of incineration. The aforementioned conditions comprise of consequences of engineered nanomaterial and the performance of these materials in ecological compartments as well as the technosphere. The dissolution of alumina nanoparticles, zinc oxide nanoparticles, and nano-silver in water enhances the harmful effects. However, the dissolution of nano-silicon dioxide reduces the harmful effects as it caused the vanishing of the nanomaterials and the dissolved silica is not dangerous at environmental concentrations. With the probable exemption of nanosilica, all other nanomaterials can agglomerate intensely in natural waters and are therefore detached from the water system and are less mobile. A majority of engineered nanomaterial seem to be separated in the course of wastewater treatment, but this is based on only limited accessible research, which have employed unrealistically higher concentrations of nanomaterials. A probable exclusion might be carbon nanotubes and silica where a lower separation rate was noted depending on functionalization. For the duration of waste incineration, the carbon-containing nanomaterials might probably be destroyed totally, while metal-oxide or metal particles might persist intact. In general, it can be concluded that mostly silver nanoparticles and zinc oxide nanoparticles could lead to a maximum risk to the environment, however the titanium dioxide nanoparticles also need to be additionally examined. From an environmental perspective and the present utilization of engineered nanomaterials like aluminum dioxide, silicon dioxide, carbon black, and montmorillonite probably cause no or little hazard to the environment (Black, 2013, [178]).

### 3.2. Health Risks of Nanomaterials from Textile Industry

**Figure 9:**
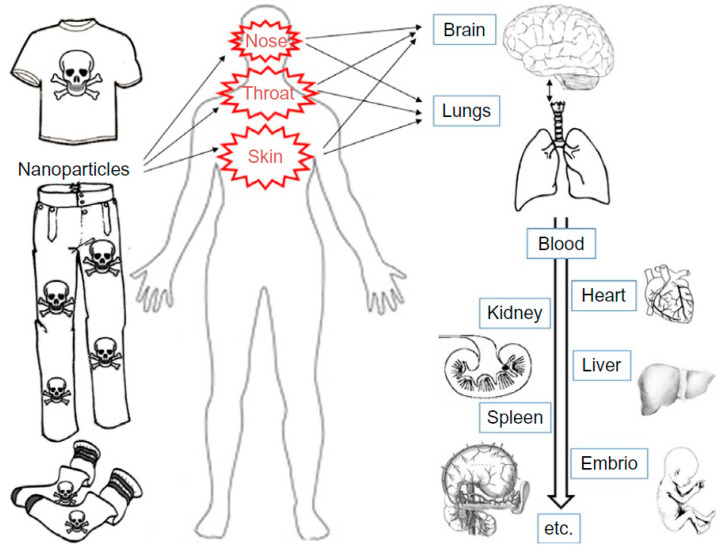
Model of the transport of nanomaterials in human body. Reproduced from (Rezic et al., 2012, [191]).

The exposure to engineered nanomaterials from textile materials could happen by means of several pathways: Skin absorption, inhalation, and ingestion (Sahu et al., 2017, [179]), (Alanezi et al., 2018, [180], (Murphy et al., 2020, [181]), (Yu, 2018, [182]), (Abdelrahman et al., 2020, [183]). Maximum exposure to nanomaterials arise for laborers in the textile industries because of continued and prolonged exposure to higher quantities of engineered nanoparticles (Torabifard et al., 2018, [184]). The most frequent routes for the engineered nanoparticle uptake are the skin as well as respiratory track. Subsequent to body entry, nanomaterial accumulates in the spleen, bon, kidney, and liver (Tavares et al., 2017, [185]). The hypothetical model of engineered nanoparticles (ENPs) pathway in the human body and its toxic and harmful effects are shown in Figure 9. Due to the aforementioned reasons, the toxico-kinetics of engineered nanomaterials are presently under numerous studies (de Jong et al., 2017, [186]). Due to the fact that the engineered nanomaterials are smaller than 100 nm, these materials could smoothly penetrate cells (Aryal et al., 2019, [187]). Even though the harmfulness of nanoparticles varies based on their properties (chemical composition, surface energy, charge, shaper, size, and others), they also depend on the living organisms and their diverse DNA covering ratios (Sukhanova et al., 2018, [188]). The major concern for nanoparticle exposure from textiles is by means of skin absorption. The skin is considered to be a superior absorptive material because of the rich supply of blood and tissue macrophages, dendrites, lymph vessels, and various types of sensory nerve endings (Ramachandran, 2014, [189]). The paper by (Filon et al., 2016 [190]) reviewed and analytically assessed evidence on the significance of different skin adsorption paths for engineered nano-objects, nanoparticles, their aggregates as well as agglomerates.

Several studies have confirmed that nanomaterials can lead to adversarial toxic effects in living beings (Exbrayat et al., 2015, [192]), (Roberto et al., 2019, [193]), and DNA damage (Grumezescu et al., 2017, [194]). Studies on DNA damage have received more research attention because of associations with cancer, neurological diseases, and ageing. In the study by (Grumezescu et al., 2017 [194]), the damage of DNA caused by different nanomaterials will be evaluated with respect to DNA damage products, types as well as detection methods. The results of the study carried out by (Nallanthighal et al., 2017, [195]) suggest that a human being with genetic polymorphisms as well as mutations in 8-Oxoguanine DNA glycosylase 1 might have enhanced susceptibility to silver nanoparticle-mediated DNA damage. The factors that regulate the possible toxicity of engineered nanoparticles are biodegradation, biodistribution, biocompatibility, inflammation, as well as interference with cells and a regular functioning of organs (Adabi et al., 2017, [196]). The aforementioned factors are associated with the reactivity, composition, shape, and size of the engineered nanoparticles.

**Figure 10:**
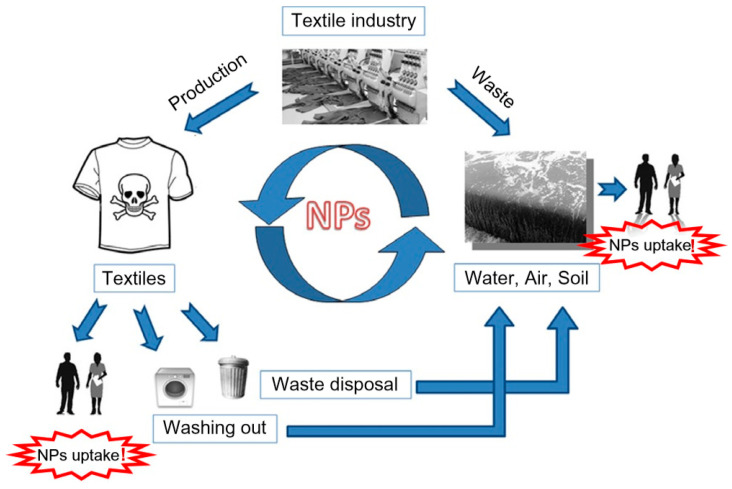
The discharge of nanoparticles (NPs) from textile materials and textile industry into surroundings and uptake by human body. Reproduced from (Yin et al., 2015, [197]).

The textiles are categorized on the basis of usage in products for babies, products with no direct skin contact, products with direct skin contact, and decorative materials. The boundaries for toxic as well as allergenic metals and chemicals differ according to the degree of fabric contact with the skin of a consumer and on heavy metal toxicity. Those limits do not involve the entire amount of compounds existing in the fabric, but the part that could be extracted (Yin et al., 2015, [197]). Analogous method must be developed for examining the harmfulness of nanoparticles on textiles, as their impact on the environment and human health is presently unpredictable. For now, humans are exposed to the emission of nanoparticles from textiles, textile industries, and textile laundries in a cycle we cannot monitor and control (Figure 10).

Therefore, the dangerous effects of nanotextiles against human health and the environment have not been broadly demonstrated yet and accessible data were extremely debatable, being contingent upon the stability, porosity, shape, size, dosage level, and end use of nanomaterials. Consequently, the obtainable reports are not much consistent and could not be compared. Further studies on the conceivable dangers in the utilization of nanofinishing in textile industries must be performed. An absence of guidelines for the control of nano-based treatments is acknowledged therefore, there might be greater concern of the supervisory establishments in the coming years. Ecolabeling is needed when nano-sized materials are included in textile clothing, particularly with direct human contact. Efforts to suggest eco-friendlier fabrication steps utilizing green chemistry, a development of nanomaterials with lesser toxicity, utilization of in-situ preparation techniques resulting in lower effluent, as well as increased durability of the nanomaterials bonded to the textile substrates will also be broadly made in forthcoming studies.

## 4. Approaches for Assessing the Nanomaterial Toxicity

The nanomaterial toxicity examinations might be carried out using in vivo (live) organisms, like rodents, fishes, microcrustaceans, and several other animals or/and cell cultures (in vitro). Different normalized toxicological examinations are obtainable for measuring the natural response of a living organism to a chemical. On the other hand, there is no standard existing for the assessment of nanomaterial toxicity, which impedes the evaluation of results as well as understanding about its toxicity. A majority of research carried out up to now are revisions of standard procedures utilized for other materials (Ju-Nam et al., 2008, [198]). Even though certain nominal associations of assays are recommended, (Drasler et al., 2017, [199]) defined that there exists no typical assessment protocol because of the extensive range of physico-chemical properties that the nanomaterials could contribute.

Animal experiments are highly prognostic for human effect however there are restrictions, primarily due to the biochemical and physiological dissimilarities among the species. Furthermore, there exist an increasing legal and public demand that morally supports the replacement of animal analysis for substitutes not based on in vivo testing. Novel concepts of testing are based on approaches with the primary culture of human cells as well as permanent cultures of cell lines, due to the fact they provide reliable, cheap, and efficient results (Drasler et al., 2017, [199]). In the following section, we discuss certain major evaluation techniques, developed both in vitro and in vivo, for properly characterizing nanomaterial toxicity.

### 4.1. In Vivo Techniques

In vitro assessments have augmented significantly, however in vivo confirmation is still needed for understanding as well as interpreting results. Additionally, animal testing was also associated with the NanoTEST venture, whose objective was to recognize the effects on the physiology of verified living organisms. Presently, the Organization for Economic Co-operation and Development (OECD) offers certain examination strategies on which biomarkers must be utilized for every testing organism (Juillerat-Jeanneret et al., 2015, [200]).

Generally, there are several studies on human toxicity by means of rodent models, while only limited research exists in vivo dealing with available nanomaterial ecotoxicity. Additionally, a majority of those observed in the studies reflect the influence of nanomaterials on aqueous organisms, due to the fact that marine and continental waters are the chief reception compartments. As a general rule, rodents (Wistar rat and mice), fish (example, Danio rerio), mollusks (example, *Lymnaea stagnalis*), microcrustaceans (example, *Ceriodaphnia dubia, D. pulex, Daphnia magna*), nematodes (example, *Caenorhabditis elegans*), algae (example, *Raphidocelis subcapitata*), and bacteria (example, *Aliivibrio fischeri*) are mostly utilized testing organisms for the assessment of acute toxicity.

### 4.2. In Vitro Techniques

As stated by (Drasler et al., 2017, [199]), assays could be carried out using eternal cell lines or primary cultures. As stated by these authors, cell lines are preferred as they provide increased stability as well as homogeneity, which favors the consistency in test results, particularly in preliminary examinations. For more precise examinations, the same investigators indorse the usage of three-dimensional co-cultures for better understanding the action mechanisms of nano-sized materials on tissues. In order to assess nanomaterial toxicity, the utilization of epithelial cell lines (lung, skin, etc.) is typically designated because the aforementioned cells demonstrate characteristics of actual barriers against destructive agents and are consequently the foremost to suffer the effect of these compounds (Rothen-Rutishauser et al., 2012, [201]). Conversely, certain strains might not be receptive to the impacts of nanomaterials and here the primary cultures might be better designated.

In the case of in vitro comet assay using the mammalian cell culture, Collins and his team members (Collins et al., 2017, [202]) put forward certain recommendations: (i) Utilize non-cytotoxic concentrations; (ii) select the type of cell as per the exposure scenario; (iii) identify both long (24 h) and short (2–3 h) tests for obtaining a clear knowledge of the action mode of the nanomaterial; and (iv) identify if the genotoxic destruction demonstrated is a consequence of the direct effect with DNA or because of DNA oxidation. As stated by (Catalán et al., 2017, [203]), the limitations as well as relevance of mutagenicity/genotoxicity assays must be considered while selecting the most suitable monitoring technique. As per the aforementioned study, examinations considered in the assessment must be based on three classes: (1) DNA damage, (2) chromosomal destruction, and (3) gene mutation. According to OECD guidelines (OECD, 2014, [204]), for selecting a test and evaluating the genotoxicity of a nanoform, the solubility, absorption, exposure, metabolites, as well as other derivatives must be taken into account, along with possible side effects.

## 5. Environmental Risk Assessment—Case Studies

In a study performed by (Voelker et al., 2015, [205]), a standard environmental risk assessment of silver nanomaterials applied in textiles has been carried out. Environmental exposure scenarios were developed for three distinct categories of textiles equipped with silver nanomaterials. Based on these scenarios, the predicted environmental concentrations were deduced for sewage treatment plants and for the environmental compartments such as surface water, sediment, and soil. The information on ecotoxicology were obtained from different analysis on earthworms, chironomids, macrophytes, duckweed, fish, daphnids, algae, cyanobacteria, activated sludge, terrestrial plants, and soil microorganisms. Emission information on silver nanomaterials NM-300K from textiles were obtained from washing experiments. The environmental risk assessment performed was based on the specifications defined in the European Chemicals Agency (ECHA) guidance on information requirements as well as chemical safety assessment. Depending on the selected scenarios as well as preconditions, no environmental hazard of the silver nanomaterials NM-300K discharged from textiles was noticed. Under conservative assumptions, a risk quotient for surface water pointed out that the marine compartment might be influenced by a higher emission of silver nanomaterials to the surroundings because of the higher sensitivity of marine life to silver. Depending on the effective retention of silver nanomaterials in sewage sludge and the continuing application of sewage sludge on farm land it is suggested to introduce a threshold for total silver concentration in sewage sludge. With regards to the potential risk mitigation measures, it is highlighted that one should directly introduce silver nanomaterials into the textile fiber due to the fact that this would lessen the discharge of silver nanomaterials extremely in the course of washing. If this is not conceivable because of technical restrictions or some other reason, then the inclusion of a threshold level controlling the discharge of silver nanomaterials from textiles is recommended. It should be noted that the aforementioned specific study is a case study that is only valid for examined silver nanomaterial NM-300K and its possible application in textiles.

**Figure 11:**
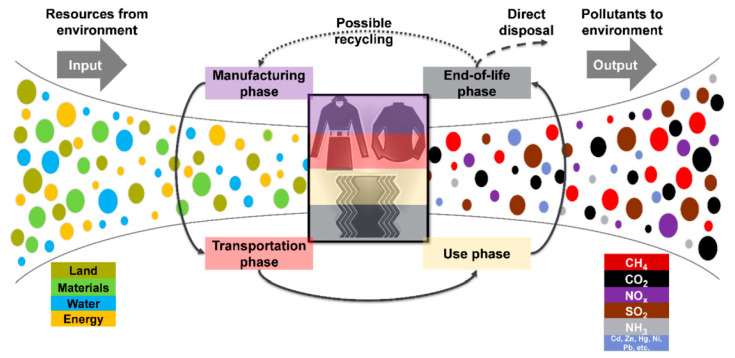
Life cycle of a textile product system as well as its environmental interventions at different phases. Reproduced from (Yasin et al., 2019, [206]).

The study conducted by (Yasin et al., 2019, [206]) elaborated on certain points for technical textile waste. Initially, the Life Cycle Assessment method for end-of-life is feasible if the waste treatment depends on the technical textile functionality instead of common textile waste. Secondly, this end-of-life study confirmed that the Life cycle assessment results of any technical textile product at its disposal are also case dependent and must not be considered the same as collective textile waste, in spite of environmental correspondence being considered or not. Figure 11 demonstrates the life cycle of a textile product system as well as its environmental interventions at different phases. The life cycle assessment “gate-to-grave” method was used for studying two technical textiles with different functionalities however with the same weight, one is a silver nanoparticle-treated polyester and the other one is flame retardant-treated wool. They were examined for having an improved understanding of environmental parity, particularly in their usage phase as well as at the “end-of-life” phase. Ten-midpoint categories were employed for analyzing the environmental impacts at the time of the use phase and end-of-life phase of both technical textiles. With regard to the technical textile curtain recycling, both use functionality substances, flame retardant (for its flame retardancy), and silver nanoparticles (for its antimicrobial properties). Their life-cycle impact perspectives can be different with their functionality lost in the application phase, for example, substantial loss of silver nanoparticles as the time of laundering, as compared to the well-bond flame retardants to the fibers. This will increase the environmental cost of one technical textile (silver nanoparticle treated) in use phase imposing severe wastewater treatment. In the same way, the behavior of other functionality substance on technical textile (flame retardant treated) needs different considerations for either end-of-life, incineration, or landfill. Thus, the overall results indicated that in the use phase, the life cycle impact of technical textiles is upfront and changes with the variation in number of washes, the types of applied attributional substances, as well as the rate of release. At the “end-of-life” phase, it has been noted that there is no relationship between the two types of technical textiles with respect to environmental impacts.

## 6. Conclusions

The state-of-the-art nanotechnology has been successfully utilized in several fields for the well-being of mankind. On the other hand, any innovative unverified technology is accompanied with certain drawbacks. There exists concerns about the conceivable hazardous impacts of nanomaterials on the environment as well as human health. There are numerous justifications for believing that the application of nanomaterials is increasing. Up until now, nanotechnology has been considerably used to develop several technologies in addition to industrial sectors, of which includes textiles. Conversely, due to the absence of suitable disposal policies, the level of nanomaterials in the surroundings is persistently developing.

Nanomaterials are promising materials for the functionalization of textiles and fibers. Nanotechnology contributes novel solutions for developing newly advanced textiles with multiple functionalities like antistatic properties, wear/tear resistance, UV protection, flame retardancy, antimicrobial property, water/oil/dirt repellency, self-cleaning ability, and wrinkle free. Despite the fact that there are still certain issues with their utilization in commercial applications, there are many substantial scientific studies that report their appropriateness for advanced functional textile applications. In addition to this progress, it should be noted that the nanotechnology field is still a developing area with several challenges as well as prospects, unsolved difficulties, and commercial opportunities. Our exposure to nanoparticles from textile materials is developing, however, unfortunately this is not supplemented with suitable awareness or/and limits prescribed by safety regulation in regards to toxicological information. It can be anticipated that this situation might vary in our immediate future. Modern science, particularly analytical chemistry with the progresses in instrumental analytics is contributing an extensive range of various techniques that could be utilized for monitoring nanoparticles that exist on textiles as well as in textile wastewaters. Research works must be carried out for preventing possible human health risks for users, inclusive of the adult population, but particularly for babies and children.

Thus, it can be concluded that nanomaterials offer both benefits as well as risks. Currently, nanomaterials are present in certain commercial products, some of which are not labelled while some are labelled, and there will certainly be more to establish. Therefore, it is significant that the textile industries have adequate knowledge about nanomaterials so as to make safe choices. The conclusions of the current paper are anticipated to increase consciousness on the possible influence of nanomaterials containing textile wastes and the significance of better regulations in regards to the ultimate disposal of these wastes.

## Figures and Tables

**Table 1:** Utilization of nanomaterials in textile functionalization.

Sl. No.	Nanomaterial	Function	Reference
1	Nanoclays	Active ingredient support, flame retardance, abrasion resistance	(Gocek et al., 2019, [77])
2	Aluminium oxide	flame retardance, abrasion resistance	(Korkmaz et al., 2016, [78])
3	Silicon dioxide	Reinforcement enhanced the dyeability, abrasion resistance, water repellence, dirt repellence	(Dogan et al., 2017, [79])
4	Zinc oxide	Stiffness, abrasion resistance, self-cleaning, antibacterial property and UV protection	(Verbic et al., 2019, [80])
5	Titanium dioxide	Water repellence, dirt repellence, self-cleaning, UV protection	(Abbas et al., 2018, [81])
6	Silver	Electrically conductive, antibacterial property	(Xu et al., 2017, [82])

**Table 2:** Textiles modified using different nanoparticles for antimicrobial effects.

Sl No.	Nanoparticles	Size	Fiber	Microorganisms	Results	Reference
1	Silver	2.3 nm	Cotton	*E. coli* and *Staphylococcus aureus*	99% reduction for Staphylococcus aureus and 92% reduction for *E. coli*	(Wu et al., 2019, [127])
2	Silver	60–100 nm	Cotton and rayon	*E. faecalis*, *S. aureus*, and *E. coli*	*E. faecalis* 97%, S. aureus-98%, and *E. coli*-100%	(Toh et al., 2017, [128])
3	Copper oxide	83 nm	Cotton	*K. pneumonaie*, *E. coli*, and *S. aureus*	Superior antimicrobial activity with antimicrobial durability of 93% subsequent to 50 washes.	(Vasantharaj et al., 2019, [129])
4	Titanium dioxide	50 nm	Cotton	*E. coli* and *S. aureus*	Greater than 95% decrease subsequent to 20 washes	(El-Naggar et al., 2016, [130])
5	Titanium dioxide -graphene oxide	Less than 100 nm	Cellulose acetate fibers	*B. cereus* and *B. subtilis*	Greater than 95% reduction	(Jia et al., 2019, [131])
